# Sex differences in the association of abdominal adipose tissue and anthropometric data with untreated hypertension in a Chinese population

**DOI:** 10.1186/s13293-020-00317-4

**Published:** 2020-07-17

**Authors:** Youzhou Chen, Zhuoli Zhang, Jihong Wang, Huayi Sun, Xingshan Zhao, Xiaoguang Cheng, Qiong Zhao

**Affiliations:** 1 Department of Cardiology, Beijing Jishuitan Hospital, No. 31 East Street, Xinjiekou, XiCheng District, Beijing, 100035 China; 2 Department of Radiology, Robert H. Lurie Comprehensive Cancer Center, Feinberg School of Medicine, Northwestern University, 737 N. Michigan Ave, 16th Floor, Chicago, USA; 3 Department of Radiology, Beijing Jishuitan Hospital, No. 31 East Street, Xinjiekou, XiCheng District, Beijing, 100035 China; 4 Inova Heart and Vascular Institute, Inova Fairfax Hospital, 3300 Gallows Road, Falls, Church, VA 22042 USA

**Keywords:** Visceral fat, Hypertension, Sex

## Abstract

**Background:**

There are inconsistent interpretations of the interrelationship of adiposity, anthropometric indices, and blood pressure (BP) in hypertensive patients. Additionally, whether these relationships differ between sexes is unknown. We aimed to elucidate the associations of adiposity indices measured using quantitative computed tomography (QCT) with BP and hypertension and to determine the effect of sex on the interrelationship of these parameters in a Chinese population.

**Methods:**

Abdominal adipose fat, including the visceral adipose tissue (VAT) area and subcutaneous adipose tissue (SAT) area, was measured by QCT in 1488 patients (514 men, 974 women). Body mass index (BMI), waist circumference (WC), hip circumference (HC), and systolic (SBP) and diastolic BP (DBP) were measured. Pearson correlation coefficients, multivariate analyses, and receiver operating characteristic (ROC) curves were used to assess the relationship and potential of adiposity indices to BP and risk of hypertension within sex groups.

**Results:**

Men had significantly greater VAT area but less SAT area than women in hypertensive group. VAT, SAT, and WC were more highly correlated with SBP in men than in women. After controlling for body weight, height, and age, VAT area and WC were positively associated with SBP (VAT: *β* = 0.309, *p* < 0.001; WC: *β* = 0.148, *p* = 0.001) and DBP (VAT: *β* = 0.099, *p* = 0.034; WC: *β* = 0.198, *p* = 0.001) in women. VAT area was positively associated with SBP (*β* = 0.444, *p* < 0.001) and DBP (*β* = 0.146, *p* = 0.021) in men. WC had a significant correlation with an increased risk of hypertension in women but a borderline association in men (*p* = 0.059) when adjusted for VAT area and SAT area.

**Conclusions:**

The association of abdominal adiposity with hypertension differs qualitatively by sex. WC may be an important determinant of hypertension and may be used for risk stratification for hypertension among Chinese individuals.

## Background

Hypertension is a common widespread disease that is associated with an increased risk of cardiovascular and cerebrovascular events [1]. Obesity, especially central obesity, is a leading risk factor for hypertension [2]. Although men and women are both susceptible to obesity, obesity-related cardiovascular mortality differs between the sexes. Men have higher cardiovascular mortality than women at each body mass index (BMI) level when adjusted for age and heart rate [3]. This may in part be related to sex differences in abdominal fat accumulation, as the distribution of fat has a greater impact on cardiometabolic risk than excess total fat mass [4].

Abdominal adipose tissue is manifested in subcutaneous adipose tissue (SAT) and visceral adipose tissue (VAT); both of which may confer differential metabolic risk profiles [5]. Previous studies have confirmed the positive relationship between abdominal adipose tissue and hypertension [6, 7]; however, whether the relationships of VAT and SAT to hypertension differ by sex remains unclear. For example, one study demonstrated a positive relationship between abdominal adipose tissue and measures of cardiometabolic risk, which were stronger in women than men [8]. Another study revealed greater VAT and a more detrimental cardiometabolic risk profile in men than in women [9]. Study samples have focused on American [8, 10] and Japanese [11] individuals, and sex differences in the effect of abdominal adipose tissue on the increased risk of hypertension are unclear in the Chinese population.

Indirect adiposity indices such as BMI, waist circumference (WC), and hip circumference (HC) are usually used as surrogate markers because these anthropometric measures are more correlated with direct adiposity measures such as VAT or SAT [7]. Increases in WC, HC, and BMI were also associated with higher risks of hypertension [12]. There are inconsistencies as to which anthropometric measures are more strongly associated with blood pressure and hypertension [13, 14]. In addition, considerable uncertainty exists regarding whether the strength of these associations differs substantially by sex.

Quantitative computed tomography (QCT) imaging techniques can accurately measure abdominal fat quality, including VAT and SAT, in vivo [15, 16]. Therefore, the purpose of this study was to explore the independent and joint association of anthropometric measures of general and central adiposity measured using QCT with various blood pressure components and hypertension and to determine the effects of sex on the relationships in a large sample of the Chinese population.

## Methods

### Study participants

The subjects of this study were from Beijing Jishuitan Hospital. This study enrolled 705 hypertensive participants (307 men, 398 women) who were not taking antihypertensive drugs and 783 healthy subjects (207 men, 576 women) for the QCT examination. The exclusion criteria were as follows: (1) subjects with infection, tumor, rheumatic immune disease, renal failure, and mental disease; (2) women who were pregnant or breastfeeding; (3) subjects with incomplete data for physical measures and blood pressure; and (4) participants who were taking statins or other lipid-lowering drugs. The study protocol was approved by the Ethics Committee of Beijing Jishuitan Hospital, and informed consent was provided by all participants.

### CT examination and QCT-derived fat measurements

**Fig. 1:**
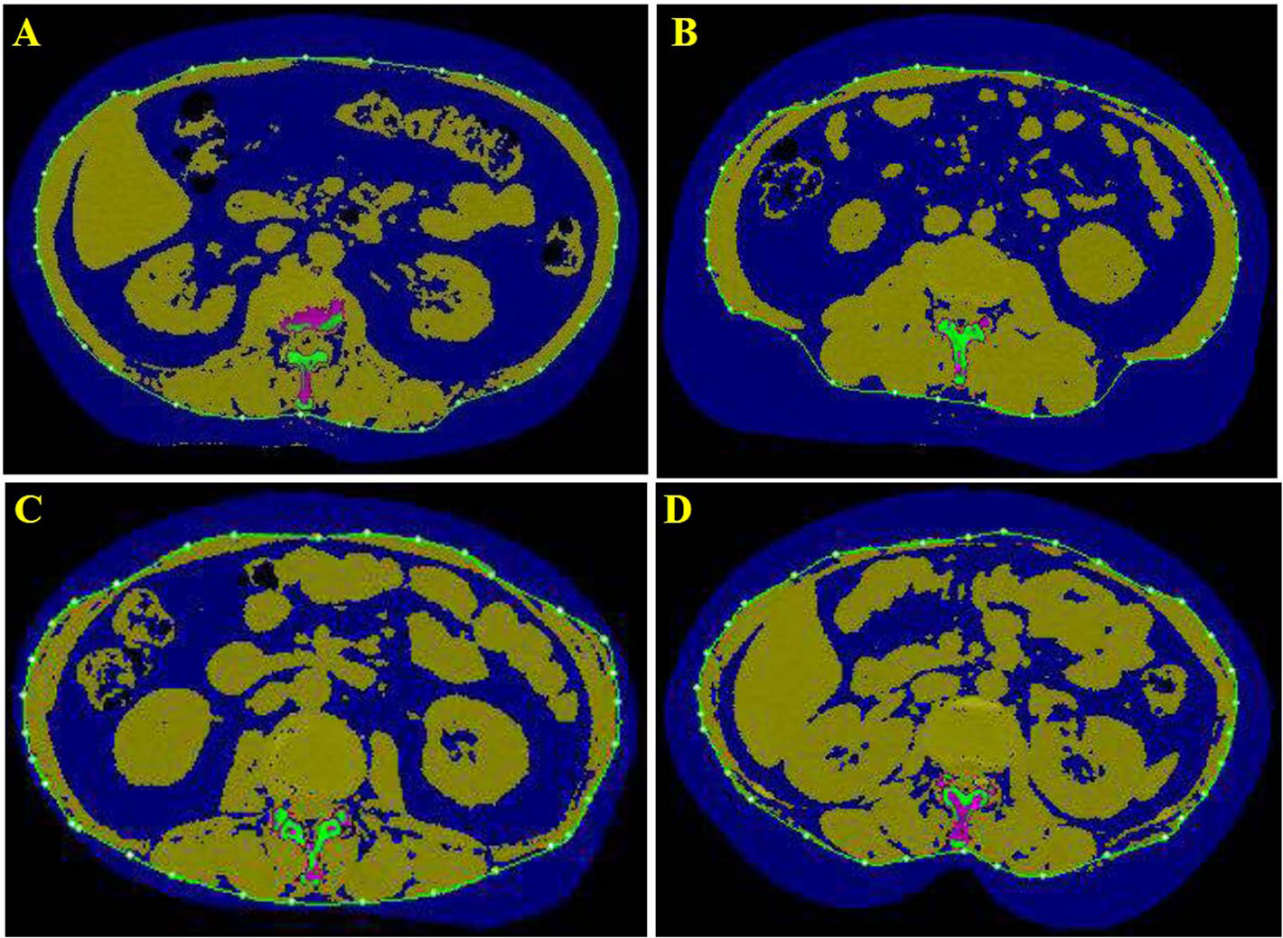
The VAT and SAT areas at the L2/3 level. The blue area in the outer layer of the green line represents the SAT area. The blue area in the inner layer of the green line represents the VAT area. VAT, visceral adipose tissue; SAT, subcutaneous adipose tissue. **a** The quantification of VAT area (304.7cm^2^) and SAT area (194.4 cm^2^) in the man with hypertension. **b** The quantification of VAT area (261.4 cm^2^) and SAT area (290.3 cm^2^) in the woman with hypertension. **c** The quantification of VAT area (146.2 cm^2^) and SAT area (76.8 cm^2^) in man without hypertension. **d** The quantification of VAT area (115.5 cm^2^) and SAT area (147.9 cm^2^) in the woman without hypertension

The details of the QCT acquisition were described previously [17]. VAT area and SAT area were measured using a Toshiba 64-slice or an 80-slice CT scanner with a QCT pro (Mindways, TX, USA) calibration phantom at the level of L2/3. The software automatically sets a closed spline at the subcutaneous margin, i.e., the boundary between the subcutaneous fat and abdominal muscle. Soft-tissue pixels were segmented on a per-pixel basis by CT value into a predicted mixture of fat and lean muscle to yield the observed CT value. The VAT area and SAT area were acquired by manually outlining the abdominal muscular wall separating the visceral from the subcutaneous fat deposits (Fig. 1). On each 1-mm-thick slice, adipose tissue was segmented and mapped in blue with the default threshold, and then the outer contour of the abdominal wall was automatically outlined by the software. Fat was defined as any voxel between − 195 HU and − 45 HU with an average attenuation of − 120 HU [18].

### Anthropometric measurements

Weight was measured in light clothing without shoes to the nearest 0.1 kg, and height was measured without shoes to the nearest centimeter. BMI was calculated by weight (kilograms) divided by the square of height (meters). WC was measured to the nearest centimeter using a tape measure at the high point of the iliac crest at minimal respiration. HC was measured to the nearest 0.1 cm around the thighs at the height of the greater trochanter in standing position.

### Blood pressure measurements

Blood pressure was measured by trained staff three times using a mercury sphygmomanometer with an appropriate cut size, after 5 min of rest, in a sitting position in a hospital between 8:00 AM and 12:00 PM. The average of these 3 consecutive measurements of BP was used in the statistical analyses. Hypertension was defined as systolic blood pressure ≥ 140 mmHg and diastolic blood pressure ≥ 90 mmHg.

### Statistical analysis

Descriptive characteristics of the normotensive and hypertensive participants are presented as the mean ± standard deviation (SD), and categorical variables are presented as cases (*n*) or percentages (%). Comparisons between groups were performed using Student’s 2-tailed *t* test for continuous variables and a chi-square test for categorical data. The Bonferroni adjustments were used for all post hoc multiple comparisons. Age-adjusted, sex-specific Pearson correlation coefficients were calculated to assess the correlations between abdominal fat volume and continuous anthropometric measures. Multivariable-adjusted linear regression models were constructed to assess the association per 1 SD increment in VAT area and SAT area with SBP and DBP levels. As previous research has identified sex differences among results [10, 15], the analysis was stratified by sex. For hypertension, two models were performed. The first model adjusted for age, BMI, HC, and WC. The second model included the same covariates as well as VAT and SAT areas. The area under receiver operating characteristic (ROC) curves was calculated to evaluate the abilities of the anthropometric indices to identify the risk of hypertension. New cutoff points were suggested by Youden’s index (sensitivity + specificity − 1). A value of *P* < 0.05 was considered statistically significant.

## Results

The characteristics and anthropometric indices of the study participants stratified by sex are presented in Table 1. The mean ages of males and females were 64.15 ± 8.43 years and 62.72 ± 8.11 years, respectively. There were no significant differences in BMI between men and women (24.17 ± 5.62 vs. 25.02 ± 4.91 kg/m^2^, *P* = 0.583). Both WC and VAT area were significantly greater in men than in women (88.69 ± 9.85 vs. 84.92 ± 9.43 cm; 170.42 ± 68.78 vs. 140.27 ± 54.21 cm^2^, respectively, *P* < 0.001). Furthermore, SBP and DBP showed the same tendency in men as in women. Compared to women, men had greater VAT area (170.42 ± 68.78 vs. 140.27 ± 54.21 cm^2^, *P* < 0.001) but lower SAT area (104.87 ± 50.27 vs. 153.26 ± 64.61 cm^2^, *P* < 0.001) (Table 1). In both men and women, the conventional anthropometric indices, such as BMI, WC, and HC, in the hypertension group were significantly greater than those in the group without hypertension. For the QCT-derived anthropometric indices, compared to hypertensive women, men had a greater VAT area but lower SAT area in the hypertension group (*P* < 0.001) (Table 2).

**Table 1:** Clinical characteristics and anthropometric indicators of adiposity in both sexes

	Total (*n* = 1488)	Men (*n* = 514)	Women (*n* = 974)	*P*
Age (year)	63.22 ± 8.25	64.15 ± 8.43	62.72 ± 8.11	0.002
Hypertension (%)	783 (52.6)	307 (59.7)	398 (40.9)	< 0.001
Height (cm)	160.47 ± 7.98	167.50 ± 6.78	156.76 ± 5.75	< 0.001
Weight (kg)	65.29 ± 13.46	71.50 ± 14.43	64.01 ± 11.74	< 0.001
BMI (kg/m^2^)	25.07 ± 5.16	24.17 ± 5.62	25.02 ± 4.91	0.583
WC (cm)	86.22 ± 9.74	88.69 ± 9.85	84.92 ± 9.43	< 0.001
HC (cm)	97.97 ± 8.58	98.57 ± 9.18	97.65 ± 8.25	0.060
VAT (cm^2^)	150.69 ± 61.32	170.42 ± 68.78	140.27 ± 54.21	< 0.001
SAT (cm^2^)	136.54 ± 64.29	104.87 ± 50.27	153.26 ± 64.61	< 0.001
SBP (mmHg)	137.71 ± 19.72	141.66 ± 18.51	135.63 ± 20.02	< 0.001
DBP (mmHg)	79.11 ± 10.93	80.45 ± 11.50	78.40 ± 10.56	0.001

*BMI* body mass index, *WC* waist circumference, *HC* hip circumference, *VAT* visceral adipose tissue, *SAT* subcutaneous adipose tissue, *SBP* systolic blood pressure, *DBP* diastolic blood pressure

**Table 2:** The anthropometric indicators of adiposity by hypertensive status

	Normal tensive group (*n* = 783)		Hypertensive group (*n* = 705)		*P* value
	Men (*n* = 207)	Women (*n* = 576)	Men (*n* = 307)	Women (*n* = 398)	
Age (year)	64.54 ± 8.05	61.51 ± 8.01^#^	63.88 ± 8.69*	64.71 ± 7.88^&^	< 0.001
Height (cm)	167.18 ± 6.09	157.01 ± 5.88^#^	167.72 ± 7.22^&^	156.40 ± 5.56	< 0.001
Weight (kg)	68.34 ± 14.01	60.45 ± 12.70^#^	73.64 ± 14.16^#&^*	64.28 ± 9.75^#&^	< 0.001
BMI (kg/m^2^)	24.19 ± 5.29	24.34 ± 5.07	25.83 ± 5.75^#&^	26.00 ± 4.48^#&^	< 0.001
WC (cm)	84.89 ± 8.52	82.59 ± 8.68^#^	91.24 ± 9.86^#&^*	88.30 ± 9.47^#&^	< 0.001
HC (cm)	96.17 ± 9.47	95.98 ± 7.69	100.11 ± 8.66^#&^	99.99 ± 8.44^#&^	< 0.001
VAT (cm^2^)	138.3 ± 50.06	124.51 ± 48.13^#^	192.20 ± 68.07^#&^*	163.09 ± 54.45^#&^	< 0.001
SAT (cm^2^)	92.40 ± 47.52	146.89 ± 62.49^#^	113.27 ± 50.40^#&^*	162.47 ± 66.57^#&^	< 0.001

*BMI* body mass index, *WC* waist circumference, *HC* hip circumference, *VAT* visceral adipose tissue, *SAT* subcutaneous adipose tissue

^#^*P* < 0.05 compared with the group without hypertension in men

^&^*P* < 0.05 compared with the group without hypertension in women

*P < 0.05 compared with the group with hypertension in women

### Correlations of VAT or SAT area with conventional anthropometric measures

Age-adjusted correlations of VAT and SAT area with conventional anthropometric measures stratified by sex are displayed in Table 3. The conventional anthropometric measures (BMI, WC, HC) were all significantly associated with VAT as well as SAT area (*P* < 0.001). Among the conventional adiposity measures, WC showed the best correlation not only with VAT area in men and women (*r* = 0.642 in men, *r* = 0.610 in women, *P* < 0.001, respectively) but also with SAT area in men and women (both *P* < 0.001).

**Table 3:** Age-adjusted Pearson correlation coefficients between simple anthropometric indices and VAT and SAT area

	Men				Women			
	VAT	*P*	SAT	*P*	VAT	*P*	SAT	*P*
BMI	0.357	< 0.001	0.313	< 0.001	0.465	< 0.001	0.480	< 0.001
WC	0.642	< 0.001	0.505	< 0.001	0.610	< 0.001	0.561	< 0.001
HC	0.453	< 0.001	0.375	< 0.001	0.521	< 0.001	0.502	< 0.001

*BMI* body mass index, *WC* waist circumference, *HC* hip circumference, *VAT* visceral adipose tissue, *SAT* subcutaneous adipose tissue

### Age-adjusted correlations of anthropometric measures with SBP and DBP

Age-adjusted correlations of anthropometric measures with SBP and DBP are shown in Table 4. The conventional anthropometric indices, such as BMI, WC, and HC, as well as VAT and SAT area were all significantly correlated with DBP and SBP (*P* < 0.001). VAT area in men showed a higher correlation with SBP than that in women (*r* = 0.469 in men, *r* = 0.388 in women). Among the conventional anthropometric indices, WC also showed higher correlations with SBP in men than in women (*r* = 0.348 in men, *r* = 0.321 in women). In contrast, compared to women, SAT area showed lower correlations with DBP in men than in women (*r* = 0.104 in men, *r* = 0.164 in women).

**Table 4:** Pearson correlation coefficients between anthropometric indices and blood pressure level after adjustment for age

	Men				Women			
	SBP	*P*	DBP	*P*	SBP	*P*	DBP	*P*
BMI	0.154	< 0.001	0.095	0.039	0.222	< 0.001	0.186	< 0.001
WC	0.348	< 0.001	0.179	< 0.001	0.321	< 0.001	0.272	< 0.001
HC	0.238	< 0.001	0.112	0.021	0.272	< 0.001	0.202	< 0.001
VAT	0.469	< 0.001	0.212	< 0.001	0.388	< 0.001	0.239	< 0.001
SAT	0.198	< 0.001	0.104	0.024	0.151	< 0.001	0.164	< 0.001

*BMI* body mass index, *WC* waist circumference, *HC* hip circumference, *VAT* visceral adipose tissue, *SAT* subcutaneous adipose tissue, *SBP* systolic blood pressure, *DBP* diastolic blood pressure

### The correlations of anthropometric indices with SBP and DBP in the linear regression model

The results of the multiple linear regression analyses for the sex-specific associations of anthropometric measures and SBP and DBP are presented in Table 5. In men, a one standard deviation increment in VAT area was positively associated with SBP (*β* = 0.444, *P* < 0.001) as well as DBP (*β* = 0.146, *P* < 0.021) after controlling for age, body weight, height, and BMI. In women, similar results were obtained, showing significant associations between VAT area and SBP (*β* = 0.309, *P* < 0.001) and DBP (*β* = 0.099, *P* = 0.034). Among conventional anthropometric measures, only WC in women was significantly associated with SBP (*β* = 0.148, *P* = 0.007) and DBP (*β* = 0.198, *P* = 0.001). In contrast to the above findings, there was an inverse association of SAT area with DBP (*β* = − 0.105, *P* = 0.009) in women, and SAT area had no significant correlation with SBP or DBP in men.

**Table 5:** The correlations of anthropometric indices with SBP and DBP levels from the multiple linear regression model

Adiposity indices	SBP		DBP	
	*β*	*P*	*β*	*P*
Men^a^				
BMI	0.004	0.989	0.176	0.532
HC	− 0.002	0.975	− 0.023	0.737
WC	0.140	0.081	0.100	0.247
VAT	0.444	< 0.001	0.146	0.021
SAT	− 0.078	0.143	− 0.020	0.724
Women^b^				
BMI	− 0.120	0.753	0.451	0.275
HC	0.051	0.315	− 0.053	0.335
WC	0.148	0.007	0.198	0.001
VAT	0.309	< 0.001	0.099	0.034
SAT	− 0.105	0.009	− 0.019	0.663

*BMI* body mass index, *WC* waist circumference, *HC* hip circumference, *VAT* visceral adipose tissue, *SAT* subcutaneous adipose tissue, *SBP* systolic blood pressure, *DBP* diastolic blood pressure

^a^In men, the *R*^2^ value in the model analysis of SBP and other covariates was 0.228; the *R*^2^ value in the model analysis of DBP and other covariates was 0.096

^b^In women, the *R*^2^ value in the model analysis of SBP and other covariates was 0.220; the *R*^2^ value in the model analysis of DBP and other covariates was 0.094

### Multivariable-adjusted regression model of hypertension and anthropometric indices

The results of the binary logistic regression analyses for the sex-specific association of hypertension with conventional anthropometric indices as well as abdominal adiposity measures are summarized in Table 6. Two models were generated in stages: the multivariable-adjusted models, with covariates including age, height, weight, HC, WC, and BMI; and a second model in which the first model was also adjusted for VAT and SAT area. In model 1, on multivariable analysis, sex was associated with the increased risk of hypertension in the Chinese population. We also found that WC was significantly associated with an increased risk of hypertension in both men (OR = 1.089, *P* < 0.001) and women (OR = 1.063, *P* < 0.001). In model 2, when additionally adjusted for VAT and SAT area, sex remained an independent determinant of the increased risk of hypertension (*P* < 0.001). VAT area correlated with an increased risk of hypertension more in men than in women (OR = 1.013 in men, OR = 1.011 in women, *P* < 0.001, respectively). WC correlated with an increased risk of hypertension only in women (OR = 1.039, *P* = 0.002). However, there was a borderline but not significant association between WC and an increased risk of hypertension (OR = 1.040, *P* = 0.059) in men. SAT area and HC were not significantly associated with an increased risk of hypertension in either women or men in the model. The optional cutoff values and corresponding sensitivity, specificity, and area under the curve (AUC) of each adiposity index for identifying the best predicted increased risk of hypertension are presented in Table 7. WC and VAT area yielded higher AUCs than BMI and HC in both women and men (*P* < 0.05). The Youden index indicated that the optimal WC cutoff value was 86.5 cm in men and 87.0 cm in women and that the optimal VAT area cutoff value was 173.77 cm^2^ in men and 138.91 cm^2^ in women. The Youden index for VAT area was highest among the measured anthropometric indices regardless of sex.

**Table 6:** Sex-specific multivariable-adjusted regressions for anthropometric indicators of adiposity with hypertension status

	All patients		Men		Women	
	OR (95% CI)	*P*	OR (95% CI)	*P*	OR (95% CI)	*P*
Model 1						
Sex	0.529 (0.410, 0.684)	< 0.001	—		—	
WC	1.075 (1.060, 1.090)	< 0.001	1.089 (1.049, 1.129)	< 0.001	1.063 (1.035, 1.093)	< 0.001
Model 2						
Sex	0.537 (0.407, 0.709)	< 0.001	—		—	
WC	1.043 (1.023, 1.063)	< 0.001	1.040 (0.999, 1.083)	0.059	1.039 [1.018, 1.078]	0.002
VAT	1.011 (1.008, 1.014)	< 0.001	1.013 (1.008, 1.017)	< 0.001	1.011 (1.007, 1.015)	< 0.001
SAT	—	0.227	—	0.712	—	0.291

Model 2, Model 1 + VAT and SAT

*WC* waist circumference, *HC* hip circumference, *VAT* visceral adipose tissue, *SAT* subcutaneous adipose tissue

**Table 7:** Comparison of ROC analyses of the association of hypertension and anthropometric indices of adiposity by sex

Variables	AUC (95% *CI*)	*P*	Cutoff	Sensitivity	Specificity	Youden Index
**Men**						
BMI (kg/m^2^)	0.654 [0.611, 0.696]	< 0.001	24.69	0.64	0.62	0.26
HC (cm)	0.658 [0.614, 0.700]	< 0.001	100.6	0.49	0.78	0.27
WC (cm)	0.706 [0.664, 0.746]	< 0.001	86.5	0.70	0.61	0.31
VAT(cm^2^)	0.732 [0.690, 0.770]	< 0.001	173.77	0.58	0.76	0.34
**Women**						
BMI (kg/m2)	0.651 [0.619, 0.681]	< 0.001	25.25	0.59	0.66	0.24
HC(cm)	0.638 [0.606, 0.668]	< 0.001	97.5	0.57	0.64	0.21
WC (cm)	0.674 [0.643, 0.704]	< 0.001	87.0	0.49	0.75	0.25
VAT(cm^2^)	0.703 [0.673, 0.732]	< 0.001	138.91	0.64	0.67	0.31

*BMI* body mass index, *WC* waist circumference, *HC* hip circumference, *VAT* visceral adipose tissue

## Discussion

In this study, significant qualitative differences by sex existed in the relationship between QCT-measured abdominal adipose tissue and hypertension. VAT area was more strongly associated with an increased risk of hypertension in men than in women. WC was more strongly related to blood pressure components than to other conventional adiposity indices, independent of BMI and VAT area. Our findings demonstrate that WC may be a more important determinant of blood pressure and hypertension and may be used for risk stratification for hypertension among Chinese individuals.

It has been documented that VAT is better associated with the presence of hypertension than SAT [8, 19, 20]. However, few studies have explored sex differences in the relative contribution of VAT and SAT to hypertension status using high-resolution QCT imaging in a large Chinese population. Our study demonstrated that VAT rather than SAT was strongly associated with an increased risk of hypertension, extending these findings in the Chinese population and highlighting the potential pathological role of visceral fat in the development of hypertension [21]. The mechanisms may be associated with the metabolically active adipose tissue in VAT, which increase sympathetic neural activity [22, 23], activating the renin-angiotensin-aldosterone system (RAAS) [24] and increasing the secretion of inflammatory cytokines [12]. In contrast, the regional distribution of SAT has a differential impact on the risk of hypertension. SAT may preferentially release more leptin, whereas VAT may secrete more inflammatory mediators [10, 25]. For example, Vega et al. reported that lower body fat correlated negatively with SBP level [26]. Consistent with their results, our study also demonstrated that SAT was negatively associated with SBP in women. Further research is needed to better understand the mechanisms that link fat distribution and cardiovascular risk.

Previous studies have demonstrated that there are sex differences in fat distribution in the study population, but the results were inconsistent [8, 27]. Fox et al. demonstrated that SAT volume is greater than VAT volume and contributes to more absolute risk in women than men [8]. Moreover, Tanaka et al. reported that only women with more visceral fat were associated with adverse cardiovascular disease profiles in normal weight white subjects [28]. However, Whitaker et al. reported that changes in the VAT/SAT ratio were greater in men than in women throughout adulthood [27]. Bidulescu et al. demonstrated that men had more VAT but less SAT in African Americans [10]. Our findings showed that VAT was more prevalent in men than women and contributed more to SBP in men than in women, which was consistent with Jiang et al.’s findings that there was a stronger association of visceral fat index with untreated hypertension in men than in women [29]. Although Tang et al. reported that men have more VAT and less SAT than women, the associations of VAT with SBP and DBP were much stronger in women than in men [21]. Because the subjects enrolled in Tang et al.’s study were aged 40 and 65, the results were not generalizable to the whole population. Men have more visceral fat than women, which may have important clinical implications due to the known correlations of VAT and cardiometabolic risk factors [8, 15, 19].

Conventional anthropometric markers such as WC, HC, and BMI were reported to be associated with higher risk of hypertension [30]. However, whether there were sex differences in the relationship between these simple markers and hypertension together with directly measured VAT and SAT has not been examined in the Chinese population. Our findings demonstrated that WC was independently associated with an increased risk of hypertension in both sexes, which was consistent with Lorbeer et al.’s result indicating that WC was also the most strongly associated marker of the anthropometric marker group [7]. Furthermore, WC was also significantly associated with hypertension in women but had a borderline correlation in men when adjusted for VAT and SAT. In contrast, in Lorbeer’s study, WC was not associated with the prevalence of hypertension after adjusting for total adipose tissue. Pausova et al. also demonstrated that WC was not associated with BP in either sex in adolescence [31].

The different results may be explained by the racial/ethnic impact on the relationship between WC and VAT [32]. For example, Carroll et al. demonstrated that white women had greater VAT than African-American women, and the relationship between WC and VAT was lower in African Americans than in Hispanics or whites [32]. Brambilla et al. demonstrated that WC can be a good predictor of VAT and that ethnicity had a significant impact on the VAT-WC relationship [33]. In our study, WC correlated more with VAT area than other conventional anthropometric measures and was independently associated with SBP and DBP in women. Our findings suggested that WC, as an appropriate surrogate for VAT, could better predict the increased risk of hypertension in the Chinese population than conventional anthropometric measures.

### Limitations and strengths

There are some limitations in our study. First, this is a cross-sectional design, which may not allow conclusions to be drawn about the cause and effect relationship between adiposity measurements and hypertension. Second, some obesity-related factors, such as lipid profiles, diet, and smoking, were not collected and therefore were not available for analysis in the present study. Third, we did not measure the total volume of VAT or SAT; however, new studies have proven that the VAT area measured at the L2/L3 level was well correlated with the total VAT volume and could feasibly be used to estimate the total VAT volume in the Chinese population [17]. Fourth, because the age range of the patients was about 55 to 71 years old in our study, whether the positive relationship between abdominal adiposity indices and hypertension also exist in other age groups (such as adolescents) will be tested in the next study. Finally, the hormonal reproductive status of women (estrogen, follicle-stimulating hormone, hormone replacement therapy) and of men (testosterone) and leptin levels were not measured and might interfere in the outcomes of the study. Future study will be needed to explore the associations between sex hormones, leptin, and the increased risk of hypertension in Chinese population.

## Conclusion

Our study provides evidence for the sex-specific relationships between anthropometric measures of abdominal adiposity and hypertension among Chinese individuals, which suggests a need for sex-specific primary prevention for obesity-related hypertension. WC, as a marker of central adiposity, may confer an increased risk of hypertension within clinically defined categories of body weight. Our data suggest that further interventional studies are warranted to test the impact of WC reduction and, in particular, the reduction of VAT on the risk of high blood pressure and cardiovascular disease.

## Perspectives and significance

It is well documented that central obesity predisposes patients to increased risk of hypertension. However, it is still unclear whether this cardiovascular risk conferred by obesity is dependent on sex. We sought to examine sex-specific associations between visceral adipose tissue (VAT) area, subcutaneous abdominal adipose tissue (SAT) area, conventional adiposity anthropometric measurements, and the increased risk of hypertension using quantitative computed tomography (QCT) in Chinese population. VAT area was significantly greater in men than in women among different body mass index (BMI) quartiles (normal weight, overweight, and obesity). In contrast, women had greater SAT area than men at every BMI level. VAT area was more strongly associated with an increased risk of hypertension than SAT in men than in women after adjustment for BMI, waist circumference (WC), and hip circumference. WC was more strongly related to blood pressure components than to other conventional adiposity indices, independent of BMI and VAT area in women. The sex-specific criteria for WC to predict the risk of hypertension was 86.5 cm in men and 87.0 cm in women. Our findings indicated that these sex-related differences should be taken into account in therapeutic approaches of preventing for obesity-related hypertension. Studies are needed to explore the contribution of sex hormones to adipose deposition as well as to the increased risk of hypertension in the future.

## Data Availability

The datasets during and/or analyzed during the current study are available from the corresponding author on reasonable request.
